# Clinical and Molecular Profiles of Treatment-Emergent Small Cell Neuroendocrine Prostate Cancers in the Era of Intensified Systemic Therapies

**DOI:** 10.1158/2767-9764.CRC-26-0370

**Published:** 2026-10-26

**Authors:** Nadeem Bilani, Jesenia M. Perez, Ana M. Aparicio, Carolina Colli-Cruz, Cora N. Sternberg, Daniela Guevara, Ella Boytim, Peter S. Nelson, Erolcan Sayar, Michael C. Haffner, Adam Sharp, Ossian Longoria, Johann de Bono, David J. VanderWeele, Justin Hwang, Emmanuel S. Antonarakis, Maha Hussain

**Affiliations:** 1 Robert H. Lurie Comprehensive Cancer Center, Feinberg School of Medicine, Northwestern University, Chicago, Illinois.; 2 Division of Hematology, Oncology, and Transplantation, Department of Medicine, Masonic Cancer Center, University of Minnesota, Minneapolis, Minnesota.; 3 Department of Genitourinary Medical Oncology, University of Texas MD Anderson Cancer Center, Houston, Texas.; 4 Englander Institute for Precision Medicine, Weill Cornell Medicine, Meyer Cancer Center, New York, New York.; 5 Divisions of Human Biology and Clinical Research, Fred Hutchinson Cancer Center, Seattle, Washington.; 6 Institute of Cancer Research, Royal Marsden NHS Foundation Trust, London, United Kingdom.

## Abstract

Variant histologic features can emerge in therapy-resistant prostate cancers, with treatment-emergent small cell neuroendocrine carcinomas (t-SCNC) being the most common. In this study, we report the clinical, histopathologic, and genomic features of 126 t-SCNCs and 14 other treatment-emergent variant histologies. This retrospective multi-institutional analysis included cases from six academic cancer centers. Molecular testing was acquired from baseline adenocarcinoma biopsies (*n* = 23) and other variant histology biopsies (*n* = 74), including 21 paired samples. A total of 140 treatment-emergent variant prostate cancers were included. Prior to variant histology detection, 48% received androgen deprivation therapy (ADT) alone, 16% received ADT plus an androgen receptor pathway inhibitor (ARPI), 15% received ADT plus chemotherapy, and 20% received triplet therapy (ADT, an ARPI, and chemotherapy). In t-SCNC, prior chemotherapy [HR, 1.75; 95% confidence interval (CI), 1.16–2.64; adjusted *P* = 0.002] or ADT plus ARPI (HR, 3.04; 95% CI, 1.73–5.33; adjusted *P* = 0.009) was associated with a shorter interval from metastatic hormone-sensitive prostate cancer diagnosis to t-SCNC detection. *TP53* alterations were found in 74% of baseline samples, significantly enriched compared with other cohorts of hormone-sensitive tumors. In paired biopsies, *RB1* alterations were markedly increased in t-SCNC compared with baseline (76% vs. 10%, *P* < 0.001). When considering combined inactivation of key tumor suppressor genes (TSG; *TP53*, *RB1*, and *PTEN*), 76% had dual loss, whereas 33% harbored alterations in all three, portending worse outcomes (HR, 2.12; 95% CI, 1.03–4.37; univariate *P* = 0.041). Prior systemic treatment exposures were associated with the timing of t-SCNC detection within this selected cohort. Deleterious *TP53* alterations were detected in three quarters of baseline tumors, with additional TSG losses (especially in *RB1*) emerging in the t-SCNC lesion.

**Significance:**

t-SCNC is an aggressive resistance phenotype lacking validated predictive or preventive strategies. In this selected cohort, baseline *TP53* alterations were common, whereas paired analyses showed increased RB1 alterations at t-SCNC detection. These findings support longitudinal molecular profiling to characterize t-SCNC evolution and identify lineage plasticity.

## Introduction

Of the various histologic subtypes of prostate cancer identified at diagnosis, prostatic acinar adenocarcinoma accounts for more than 95% of cases (1). Although *de novo* small cell carcinoma of the prostate is rare, treatment-emergent small cell neuroendocrine carcinoma (t-SCNC) is increasingly detected in the last decade of systemic therapy intensification of metastatic hormone-sensitive prostate cancer (mHSPC; refs. 2–5). The current standard therapy for mHSPC is androgen deprivation therapies (ADT) in combination with androgen receptor pathway inhibitors (ARPI), with or without upfront docetaxel chemotherapy. Although these therapies have led to significant improvements in overall survival (OS), potent androgen receptor (AR)–directed and cytotoxic therapies have been hypothesized to impose selective pressures that may contribute to the emergence of lineage plasticity phenotypes, including t-SCNC (2–5). Examining patient-matched samples from initial adenocarcinoma and subsequent variant histology may therefore help characterize clinical and genomic features associated with the evolution and timing of t-SCNC. Preclinical mechanistic investigation is critically needed.

Clinically, t-SCNCs should be suspected in patients with mHSPC who develop rapidly progressive disease, often with visceral (liver, lung, and brain) or osteolytic bone metastases, bulky tumor masses, and disproportionately low or undetectable prostate-specific antigen (PSA; refs. 2, 6). However, definitive diagnosis requires a biopsy of a metastatic lesion. Histologically, t-SCNC is characterized by small cell carcinoma morphology and high mitotic rates (7); frequent expression of neuroendocrine markers, including synaptophysin, chromogranin A, and INSM1; and often loss of AR expression (8–10). A subset of t-SCNC cases may lack detectable neuroendocrine marker expression (11). There are no established, guideline-endorsed mechanisms to screen for the emergence of small cell carcinoma in prostate cancer. The American Society of Clinical Oncology (ASCO) recommends that the optimal first-line treatment for small cell carcinomas with prostate tumor origins is platinum-based chemotherapy with either cisplatin or carboplatin plus etoposide or with cabazitaxel, as well as the consideration of immunotherapy (12). This recommendation is based on the management of small cell lung cancer, as well as observations in randomized phase II trials in men with aggressive variant prostate cancers encompassing t-SCNC, due to the lack of phase III randomized prospective trials in prostate cancer (13, 14). After progression on platinum-based chemotherapy, enrollment in clinical trials is recommended, as there is no established second-line standard of care. Other options, such as delta-like ligand 3 (DLL3)–targeted immune engagers (NCT06117774), have been considered although limited responses have been shown to date (15).

Mechanistically, the emergence of t-SCNC has been reported preclinically, and some of these findings are evident in clinical specimens. There is consensus that this may be associated with the loss of AR activity and epigenetic reprogramming that drives a neuroendocrine prostate cancer (NEPC) transcriptome (2, 3, 16, 17). This explains, in part, the disproportionate PSA levels relative to disease volume, as *KLK3*/PSA is a direct transcriptional target of the AR. The loss of AR activity is associated with an increase in the transcriptional activity of *SOX2*, *ASCL1*, *MYCN*, *MYCL*, *EZH2*, and *POU3F2* (18–21). Certain kinases, such as *AURKA* and *RET*, have also been found to be upregulated in t-SCNCs (22, 23). In mouse models, the loss of tumor suppressor genes (TSG), including *Trp53* and *Rb1*, are initiating events, and the human homologs *TP53* and *RB1* are commonly altered in human t-SCNCs (2–4, 24).

In this study, we assembled a multi-institutional consortium to systematically investigate this aggressive disease entity based on clinical and molecular features. The cohort includes 126 t-SCNCs and 14 other histologic variants. Next-generation sequencing (NGS) results from adenocarcinoma biopsies (*n* = 23) and variant histology biopsies (*n* = 74) were analyzed. Changes from baseline tissue sampling and after variant histology sampling were studied in 21 cases with paired NGS. To our knowledge, this represents the largest reported cohort to date with clinical and molecular data from the pre- and postemergence of variant histology.

## Materials and Methods

### Patient samples

We collated case series data retrospectively through electronic medical record review from six institutions. Institutional Review Board (IRB) approval or exemption was obtained at each participating site prior to data collection, as applicable. Patients were eligible for inclusion if they had a confirmed initial diagnosis of prostate adenocarcinoma and subsequently developed t-SCNC or other variant histologies, as documented by the institutional pathology report. We included other histologic variants of prostate cancer, hypothesizing that these may have similar molecular and clinical features to classical t-SCNC variants. Due to the retrospective nature of this case series, there was no prospective enrollment, intervention assignment, or patient dropout after enrollment. Study attrition was defined as exclusion from the analytic cohort after screening because of failure to meet eligibility criteria, duplicate records, absent pathologic confirmation, or insufficient clinical data. Additionally, no formal *a priori* power calculation was performed. The sample size was determined by the number of eligible cases identified across the six participating institutions during the retrospective ascertainment period.

Demographic, clinical, pathologic, and treatment data were abstracted at each center using a standardized data collection template. Variables included age at initial diagnosis and at the time of treatment-emergent variant histology identification, race/ethnicity, comorbidities (including autoimmune disease, chronic infections, and other immunodeficiencies), and the anatomic site of variant histology biopsy. Because the study population consisted of patients with prostate cancer and was expected to be overwhelmingly composed of individuals with prostate glands, sex as a biological variable was not formally evaluated as a comparative exposure. No eligibility restriction was made on gender identity. Clinical parameters included Gleason score, PSA levels at key time points, treatment history (including types and timing of hormonal therapy, chemotherapy, radiotherapy, and radiopharmaceutical therapy), and imaging findings when available. PSA trajectory at the time of treatment-emergent histology detection was assessed relative to diagnosis as well as the PSA nadir preceding variant histology detection. PSA progression was defined as an increase of >25% above nadir; patients not meeting this threshold were categorized as having stable or declining PSA based on serial PSA measurements. Pathologic features were extracted from original biopsy and surgical resection reports and from the metachronous metastatic tumors. No randomization was performed because this was an observational retrospective case series. Cohort assignment was based on the observed treatment-emergent histology documented in the institutional pathology report. For this retrospective analysis, bias reduction strategies included the use of prespecified eligibility criteria, a standardized data abstraction template, harmonized variable definitions, and analysis after cohort definitions were finalized. Data abstractors and analysts were not blinded to the histologic cohort because histology was the defining exposure of interest.

### Histopathologic classification

The primary study cohort designation of t-SCNC versus other treatment-emergent variant histologies was based on the original pathologic diagnosis rendered at the participating institution, interpreted in conjunction with the patient’s overall clinical assessment by the treating medical oncologist. This represented the working clinicopathologic diagnosis upon which subsequent oncologic management was based. Mixed tumors were included within the original t-SCNC cohort when the institutional pathologic diagnosis and overall clinical assessment supported that designation; no minimum percentage of small cell components was imposed retrospectively for primary cohort assignment. Neuroendocrine marker expression, AR/PSA loss, Ki-67, and other IHC findings were considered supportive when available rather than universally required criteria for primary cohort assignment. No centralized pathology rereview was performed for the purposes of primary cohort assignment. To better characterize the spectrum of NEPC and other treatment-emergent variant disease represented within the cohort, participating institutional pathologists subsequently performed a retrospective secondary review of the treatment-emergent specimens. Participating sites were asked to classify the treatment-emergent biopsy based on the integrated pathologic assessment, including tumor morphology and, when available, IHC findings, proliferative index (Ki-67), and other pathologic features. For this secondary review, cases lacking a clear pathologic diagnosis of small cell morphology were not classified as small cell carcinoma solely on the basis of an AR-negative, neuroendocrine marker–positive immunophenotype. Specimens were categorized as follows: (i) small cell carcinoma, (ii) high-grade neuroendocrine carcinoma without definitive classical small cell morphology, (iii) adenocarcinoma with neuroendocrine features or a neuroendocrine component, (iv) amphicrine carcinoma, (v) large cell or giant cell carcinoma, or (vi) other variant histologies, including squamous carcinoma and carcinosarcoma. This secondary histopathologic classification was used descriptively to characterize the heterogeneity of the cohort and did not alter the original t-SCNC versus other variant cohort assignments used for the primary clinical and molecular analyses. Findings from this secondary review are summarized in Supplementary Table S1.

### Molecular testing

Genomic data from tumor tissue at initial diagnosis and from tissue confirming treatment-emergent variant histology were collected. All genomic analyses in this study were derived from tumor tissue. Circulating tumor DNA assays were not used for molecular profiling or included in the reported analyses. Genomic testing platforms included institutional (academic) or commercial NGS panels, summarized in Supplementary Table S1. Pathogenic variants, copy-number calls, variant allele frequencies (VAF), or additional reporting information was extracted through the test reports and not raw sequencing data. For all genes and alteration classes included in Figs. 2–4, the relevant gene and alteration type were evaluable by the assays contributing to those analyses. Gene-specific denominators, therefore, included only samples in which the relevant gene and alteration type were assessable. Variant classification and alteration calls were used as reported by the respective clinical assay. Differences in analytic sensitivity among platforms were not recalibrated because raw sequencing data were not uniformly available.

### Statistics

Descriptive statistics were used to characterize the study population. Categorical variables were summarized using frequencies and proportions, whereas continuous variables were reported as means with SD for normally distributed variables and medians with interquartile ranges (IQR) for non-normally distributed variables. The prevalence of gene alterations across cohorts was assessed using the Fisher exact test and McNemar test for patient-matched samples. The Wilcoxon test was used to assess VAF, tumor mutational burden (TMB), genomic loss of heterozygosity (gLOH), and programmed cell death ligand 1 (PD-L1) expression of matched patient samples. Univariate Cox proportional hazards models and Kaplan–Meier analyses were used to assess the time from mHSPC diagnosis to treatment-emergent histology identification and the time from treatment-emergent histology identification to death. For time to variant histology identification, events occurring within 5 years of mHSPC diagnosis were modeled. Because the analytic cohort was restricted to patients who developed treatment-emergent variant histology and did not include a comparator population without t-SCNC, these analyses evaluated factors associated with the relative timing of variant histology detection within the selected cohort and were not designed to estimate the incidence or risk of t-SCNC transformation. Multivariate analysis was used to establish adjusted *P* values based on age, race, ethnicity, and log_2_ (PSA at diagnosis). All analyses and visualizations were conducted using R (v4.4.1).

### Ethics statement

This study was conducted in accordance with the guidelines of the Declaration of Helsinki, Belmont Report, and U.S. Common Rule. IRB approval or exemption was obtained at each participating institution, as applicable. In keeping with 45 CFR 46.104(d)(4), the study utilized retrospective, deidentified clinical data. Written informed consent was not obtained because the requirement for informed consent was waived or deemed not required by the applicable IRB or ethics committee.

## Results

### Demographics of the cohort of t-SCNC and other histologic variants

Patients were included based on an original institutional clinicopathologic designation of t-SCNC (*n* = 126) or other treatment-emergent variant histology (*n* = 14) following an initial diagnosis of prostate adenocarcinoma. Of the other 14 tumors, mixed neuroendocrine histologies with adenocarcinoma, amphicrine cells, large and giant cell morphologies, or carcinosarcoma were reported. Records from six cancer centers (MD Anderson Cancer Center, *n* = 81; Weill Cornell Medicine/Englander Institute for Precision Medicine, *n* = 28; University of Minnesota, *n* = 14; Fred Hutchinson Cancer Center, *n* = 8; Institute of Cancer Research/Royal Marsden, *n* = 6; and Northwestern University/Lurie Cancer Center, *n* = 3) were reviewed to construct this retrospective case series (Table 1). Selected additional cohort characteristics are summarized in Supplementary Tables S1 and S2. Retrospective secondary pathology review demonstrated a spectrum of treatment-emergent morphologies, including small cell carcinoma, high-grade neuroendocrine carcinoma without definitive classical small cell morphology, adenocarcinoma with neuroendocrine features or a neuroendocrine component, amphicrine carcinoma, large- or giant-cell carcinoma, and other variant histologies. The distribution of these secondary histopathologic classifications is summarized in Supplementary Table S1. In addition, the anatomic distribution of treatment-emergent biopsy sites is also summarized in Supplementary Table S1.

**Table 1.** Cohort demographics.

Variable^a,b^	t-SCNC *N* = 126	Other variants *N* = 14	Overall *N* = 140
Age at diagnosis (years)	63 (57, 69)	62 (58, 67)	63 (57, 69)
Race			
Asian	2 (1.6%)	0 (0%)	2 (1.5%)
Black	14 (11%)	1 (7.1%)	15 (11%)
White	97 (79%)	13 (93%)	110 (80%)
Other	7 (5.7%)	0 (0%)	7 (5.1%)
Declined	3 (2.4%)	0 (0%)	3 (2.2%)
Ethnicity			
Hispanic	11 (8.9%)	0 (0%)	11 (8%)
Non-Hispanic	111 (90%)	14 (100%)	125 (91%)
Declined	1 (0.8%)	0 (0%)	1 (0.7%)
Clinical T stage			
1	4 (7.3%)	0 (0%)	4 (6%)
2	15 (27%)	1 (8.3%)	16 (24%)
3	30 (55%)	10 (83%)	40 (60%)
4	6 (11%)	1 (8.3%)	7 (10%)
Clinical N stage			
0	32 (52%)	5 (45%)	37 (51%)
1	29 (48%)	6 (55%)	35 (49%)
Clinical M stage			
0	34 (48%)	9 (75%)	43 (52%)
1	37 (52%)	3 (25%)	40 (48%)
Grade group			
1	12 (11%)	0 (0%)	12 (9.8%)
2	9 (8.3%)	1 (7.1%)	10 (8.2%)
3	16 (15%)	4 (29%)	20 (16%)
4	12 (11%)	2 (14%)	14 (11%)
5	59 (55%)	7 (50%)	66 (54%)
PSA at diagnosis (ng/mL)	12 (6, 46)	14 (6, 50)	12 (6, 48)
PSA at treatment-emergent histology detection (ng/mL)	1 (0, 17)	2 (0, 20)	1 (0, 18)
PSA trend compared with diagnosis			
Increase	25 (20%)	2 (14%)	27 (19%)
Decrease	92 (74%)	12 (86%)	104 (75%)
No change	8 (6.4%)	0 (0%)	8 (5.8%)

a Values are median (Q1, Q3) for continuous variables and *n* (%) for categorical variables.

b Percentages are calculated among patients with nonmissing data for each variable; denominators therefore vary.

Although the two histologic subsets were of disproportionate size, we did not identify clear demographic differences between the two groups when we examined age at diagnosis or race/ethnicity. At diagnosis, the majority of tumors presented with locally advanced disease (T3 and T4), whereas approximately half exhibited nodal involvement and metastatic disease. The majority of the tumors were grade group 3 or above at the time of diagnosis. The median PSA levels at initial diagnosis were 12 and 14 ng/mL for the t-SCNC and other variant groups, respectively, and remained relatively low at the time of treatment-emergent histology detection at 1 and 2 ng/mL, respectively. Among 127 patients with evaluable PSA trajectory relative to nadir, 43 (33.9%) demonstrated PSA progression, defined as an increase of >25% above nadir, whereas 84 (66.1%) did not demonstrate PSA progression, including 47 (37%) with declining PSA and 37 (29.1%) with stable PSA. PSA trajectory was unavailable for 13 patients (Supplementary Table S1). Separately, when PSA at treatment-emergent histology detection was compared with PSA at initial diagnosis, 104 of 139 evaluable patients exhibited decreases, 27 exhibited increases, and 8 had no change (Supplementary Fig. S1A). The median time from mHSPC to treatment-emergent histology detection was 33.9 months (IQR, 19.5–88.7 months; Supplementary Table S2). Lastly, we determined that based on time to treatment-emergent histology identification, t-SCNCs and other variants did not exhibit significant differences (Supplementary Fig. S1B).

### Association with systemic therapies

Given the potential implication that systemic ARPI or chemotherapies are associated with the development of t-SCNCs or other variants (2–5), we examined the therapies for mHSPC prior to emergence of variant histology (Table 2). Prior to the detection of treatment-emergent histologies, nearly all patients had received ADT (99%). When considering the two groups of variants together, 65 (48%) patients received ADT alone, 22 (16%) received ADT plus an ARPI, 47 (35%) received ADT plus chemotherapy, and 2 (1.5%) received other therapy prior to transformation (Table 2). After the detection of t-SCNC, only 17 (12%) continued to receive hormone therapies, and 34 (24%) received investigational drugs, whereas most patients received chemotherapies (*n* = 97, 69%). More detailed treatment histories before treatment-emergent histology detection are provided in Supplementary Table S2. Initial therapy included ADT plus ARPI in 35 patients (25%), ADT plus chemotherapy in 5 (3.6%), and triplet therapy with ADT, ARPI, and chemotherapy in 6 (4.3%). During subsequent pretransformation treatment, 23 patients (16.4%) received ADT plus ARPI, 1 (0.7%) received ADT plus chemotherapy, and 9 (6.4%) received triplet therapy. Radiation exposure was documented in 24 patients (17.1%) during initial treatment and 14 (10%) during subsequent pretransformation treatment. Radiopharmaceutical exposure was not documented during initial treatment and was reported in eight patients (5.7%) during subsequent pretransformation treatment (Supplementary Table S2).

**Table 2.** Treatment information.

Timing relative to treatment-emergent histology detection^a^	Treatment category	t-SCNC *N* = 126	Other variants *N* = 14	Overall *N* = 140
Prior to detection^b^	ADT alone	65 (53%)	0 (0%)	65 (48%)
​	ADT + ARPI	19 (16%)	3 (21%)	22 (16%)
​	ADT + chemotherapy (with or without ARPI)^c^	37 (30%)	10 (71%)	47 (35%)
​	Other	1 (0.8%)	1 (7.1%)	2 (1.5%)
After detection^d^	Chemotherapy	96 (76%)	1 (7.1%)	97 (69%)
​	Hormone therapy	16 (13%)	1 (7.1%)	17 (12%)
​	Investigational/targeted therapy	32 (25%)	2 (14%)	34 (24%)

a Values are *n* (%).

b For treatment categories prior to detection, percentages are calculated among patients with available category data (t-SCNC, *n* = 122; other variants, *n* = 14; overall, *n* = 136); 4 patients with t-SCNC had missing treatment category data.

c The ADT + chemotherapy category includes regimens with or without an ARPI.

d Postdetection treatment categories are not mutually exclusive.

**Figure 1.**
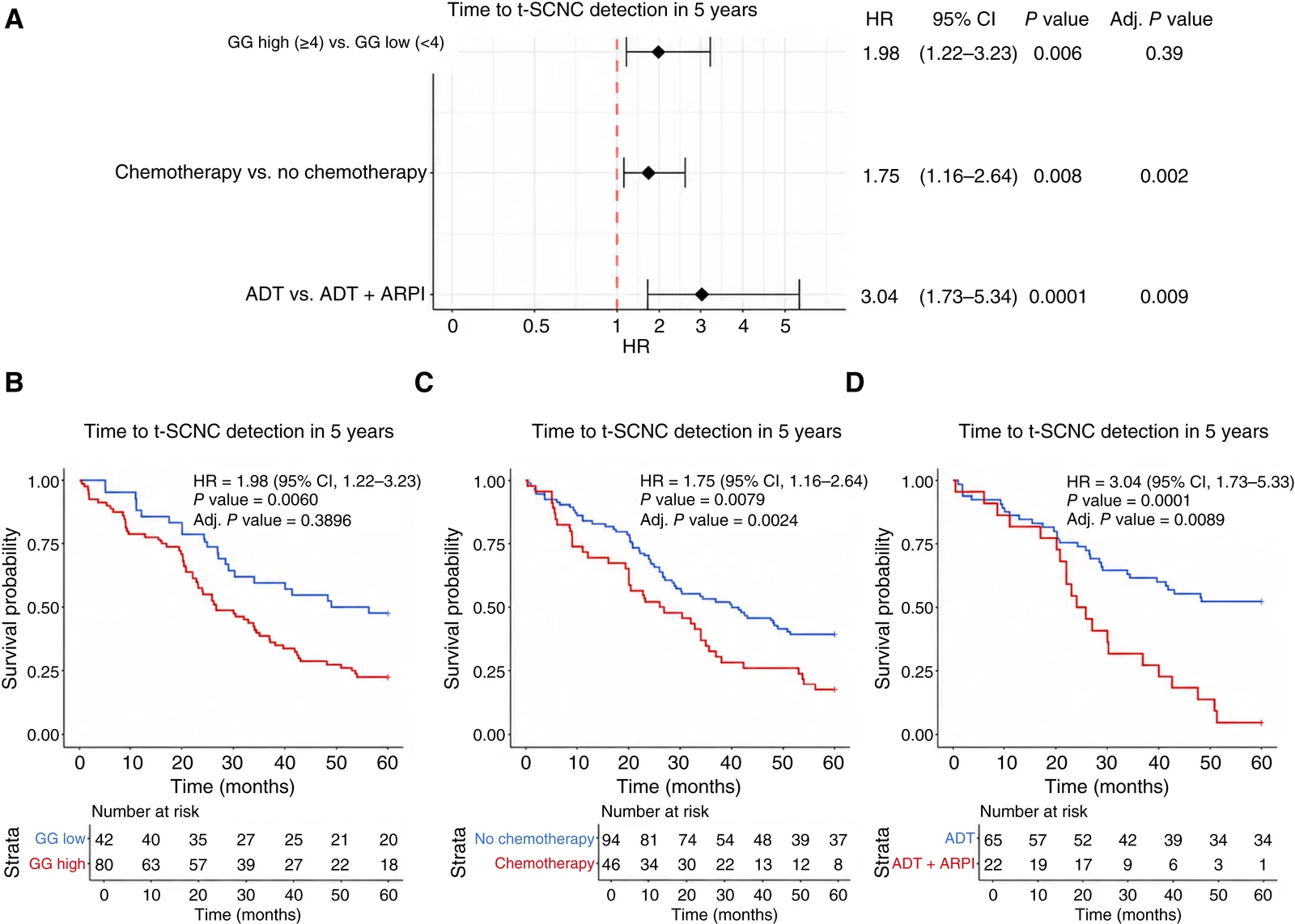
Clinical factors associated with time to t-SCNC detection. **A,** Forest plot of Cox proportional hazards models evaluating clinical factors associated with time to t-SCNC detection, including primary tumor grade group (GG), prior chemotherapy, and ADT plus an ARPI. Kaplan–Meier estimates of time to t-SCNC detection according to (**B**) primary tumor GG (≥4 vs. <4), (**C**) receipt of chemotherapy prior to t-SCNC detection, and (**D**) receipt of ADT plus ARPI prior to t-SCNC detection.

Although our cohort was selected and does not represent the prevalence of t-SCNC in the broader prostate cancer population, these treatment histories demonstrate that treatment-emergent variant histology was observed following a range of prior systemic treatment exposures. We therefore used Cox proportional hazards models and Kaplan–Meier analyses to evaluate whether baseline grade group and prior systemic treatment exposures were associated with the interval from mHSPC diagnosis to treatment-emergent histology detection within this cohort (Fig. 1A). In univariate analysis, high-grade group (≥4 vs. <4) was associated with a shorter interval to detection [HR, 1.98; 95% confidence interval (CI), 1.22–3.23; *P* = 0.006] although this association was not significant after multivariable adjustment (adjusted *P* = 0.390; Fig. 1B). Prior chemotherapy was associated with a shorter interval to t-SCNC detection (HR, 1.75; 95% CI, 1.16–2.64; *P* = 0.008; adjusted *P* = 0.002), as was ADT plus ARPI exposure (HR, 3.04; 95% CI, 1.73–5.33; *P* = 0.0001; adjusted *P* = 0.009; Fig. 1C and D). These analyses describe differences in the timing of detection among patients within this selected cohort and do not estimate the risk of developing t-SCNC.

### Molecular associations in baseline and variant histology samples

**Figure 2.**
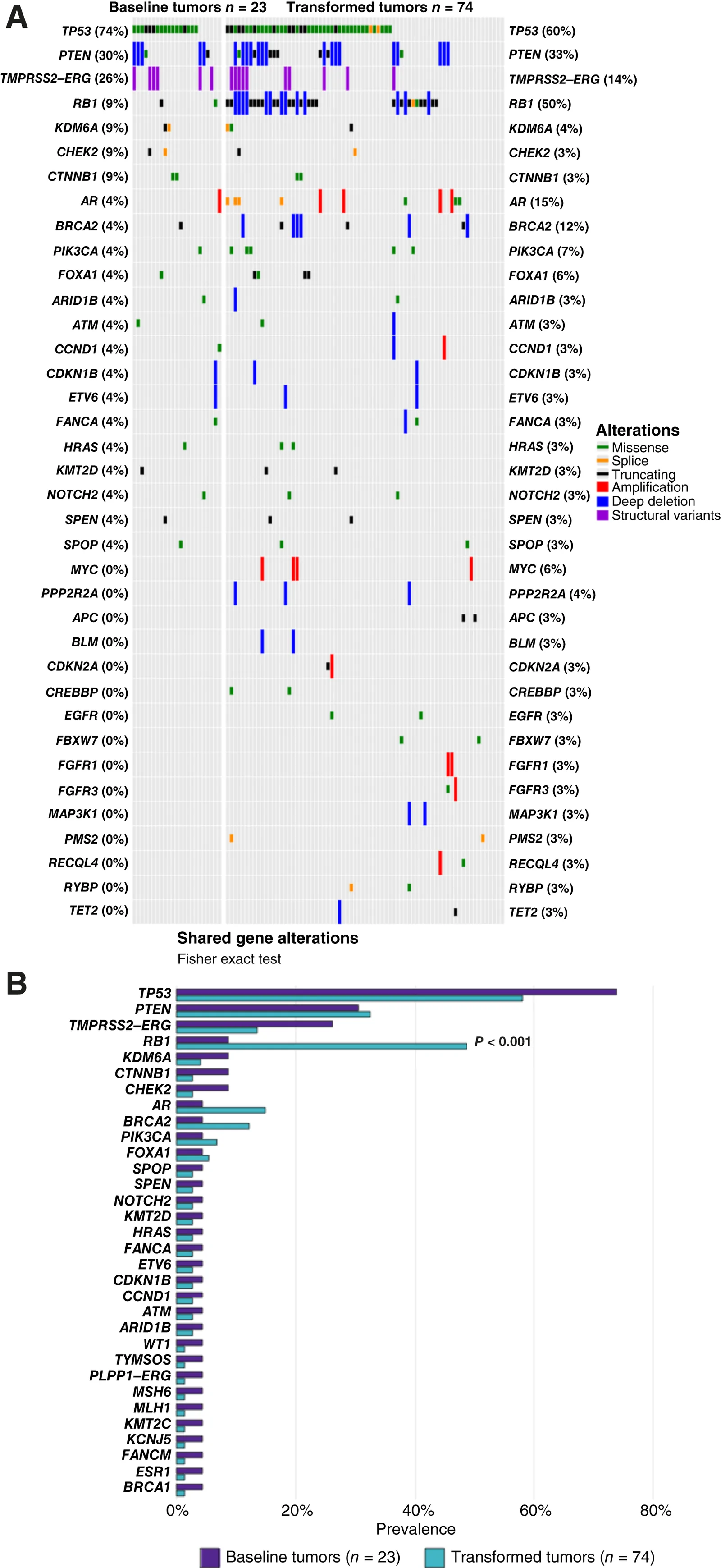
Genomic alterations in baseline and treatment-emergent tumor specimens. **A,** OncoPrint depicting pathogenic genomic alterations identified in baseline adenocarcinoma and treatment-emergent tumor specimens. Alterations include missense mutations, splice variants, truncating mutations, amplifications, deep deletions, and structural variants. **B,** Prevalence of recurrent genomic alterations in baseline and treatment-emergent specimens. The Fisher exact test was used to compare alteration prevalence between groups.

Based on genomic testing results, we examined the prevalence of pathogenic alterations in both the adenocarcinoma and variant histology tumors (Fig. 2A). Pathogenic alterations encompass missense mutations, splice variants, truncating mutations, amplifications, deep deletions, and structural variants. Although the testing platforms we used differ from the research grade used in The Cancer Genome Atlas (TCGA), we noted that the 23 baseline tumors harbored expected rates for genes recurrently altered in prostate cancer, including *PTEN* (30%) and *TMPRSS2-ERG* (26%). Only 1 baseline tumor harbored an AR amplification. Of these baseline samples, *TP53* alterations were detected in 74%. This exceeded the primary tumor detection rate in TCGA (16%), as well as the Stand Up 2 Cancer (SU2C) study prevalence that examined 444 metastatic castration-resistant prostate cancers (mCRPC; ref. 25), in which 40% harbored *TP53* alterations. In real-world patients, prior studies found that 35.4% (*n* = 840) of ADT-naïve patients presented with TP53 alterations, and this increased to 51.5% (*n* = 330) in mCRPCs (26). Other than baseline *TP53* alterations, we also noted that the t-SCNC tumors harbored fivefold more *RB1* alterations (9%–50%, *P* < 0.001). This was the only genomic alteration that exhibited a significant change in prevalence when comparing the pretreatment adenocarcinomas with the t-SCNC tumors (Fig. 2B). This also exceeded the frequency found in reported real-world mCRPC cohorts (7.9%, *n* = 330; ref. 26). Of these *RB1* alterations, we found functionally impactful mutation types including missense, truncating, splice sites, and deep deletions (Supplementary Fig. S2). When considering the enrichment of these specific types of alterations, none were independently significant.

**Figure 3.**
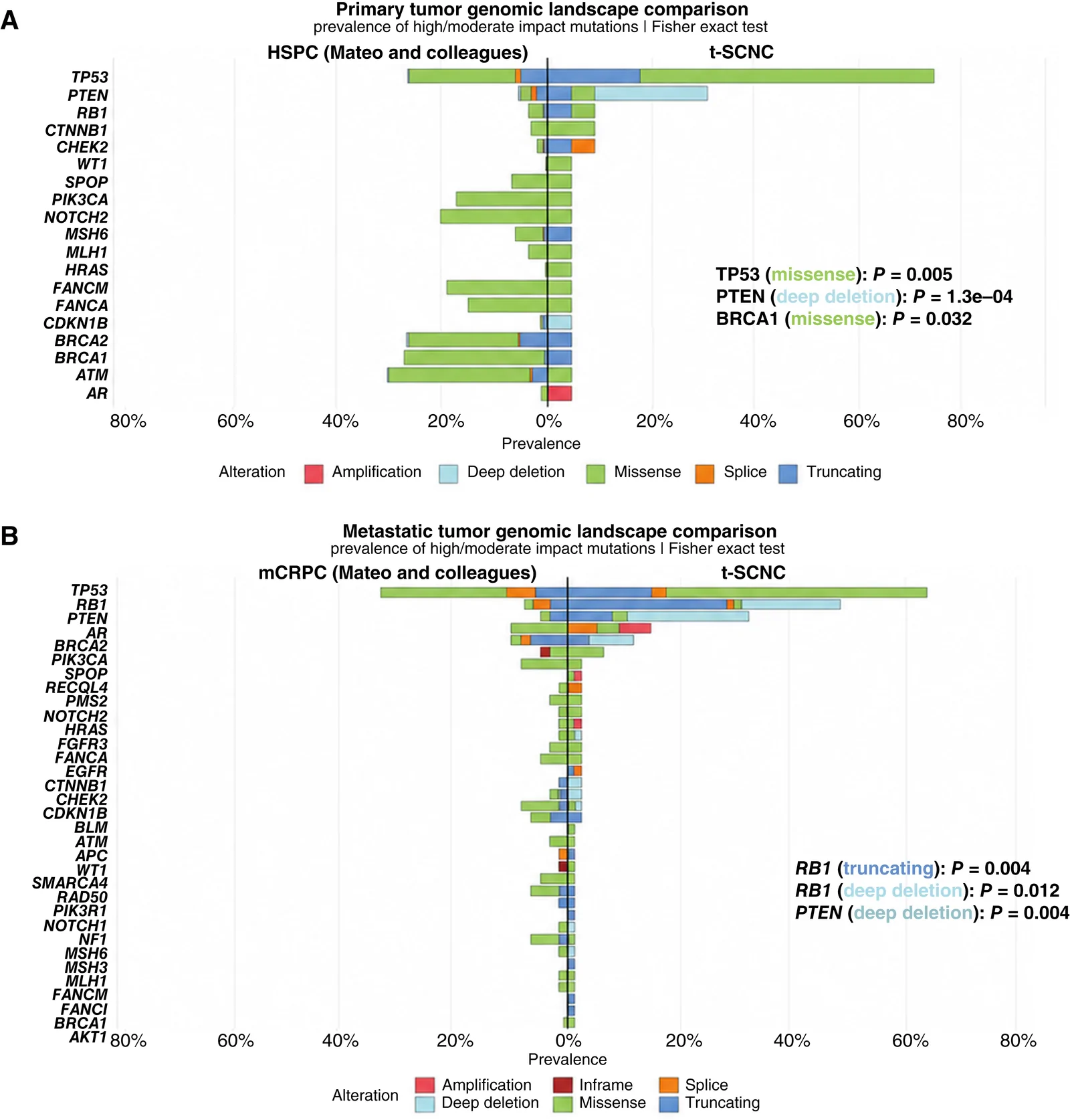
Genomic alterations in the study cohort compared with the Mateo and colleagues prostate cancer cohort. Genomic alteration frequencies in the present cohort were compared with those reported by Mateo and colleagues. **A,** Comparison of baseline specimens from the present cohort with treatment-naïve specimens from the Mateo and colleagues cohort. **B,** Comparison of treatment-emergent specimens from the present cohort with progression specimens from the Mateo and colleagues cohort. Fisher exact tests were used to evaluate differences in alteration prevalence.

To further evaluate the importance of these genomic aberrations toward the development of t-SCNC, we examined a parallel study by Mateo and colleagues (27), in which 470 treatment-naïve patients were evaluated. However, in contrast to our cohort, the 52 progression samples retained an adenocarcinoma phenotype at the time of mCRPC progression rather than the emergence of t-SCNC or other variant histologies. Here, we sought to compare patterns of genomic evolution from mHSPC to adenocarcinoma mCRPC with those from mHSPC to t-SCNC. When comparing the baseline samples, we note that our t-SCNC cohort was enriched for missense *TP53* mutations and *PTEN* deep deletions but had fewer *BRCA1* missense mutations (Fig. 3A). Furthermore, when we examined the t-SCNC samples, we note that our cohort exhibited an increased prevalence of truncating or deep deletions of *RB1* and deep deletions of *PTEN* (Fig. 3B). Given the enrichment of *TP53* alterations, we examined whether there were differences in dominant-negative (DN) *TP53* mutations (R175, R248, R273, R282, and G245), which have been reported to portend worse outcomes in mHSPCs compared with other loss-of-function variants (28). Here, whether we examined our entire molecularly characterized cohort or the paired samples, *TP53* DN mutations exhibited increased prevalence in t-SCNC samples compared with the Mateo and colleagues cohort (Supplementary Fig. S4). Altogether, these cross-cohort comparisons demonstrate the enrichment of *TP53* alterations in our t-SCNC cohort and support further investigation of *TP53* alterations as a potential biomarker of subsequent t-SCNC. Furthermore, compared with a cohort that develops mCRPCs, our cohort also harbored differences in key TSGs, including *TP53*, *RB1*, and *PTEN*, in both the baseline and treatment-emergent samples.

### Molecular interrogation of paired samples

**Figure 4.**
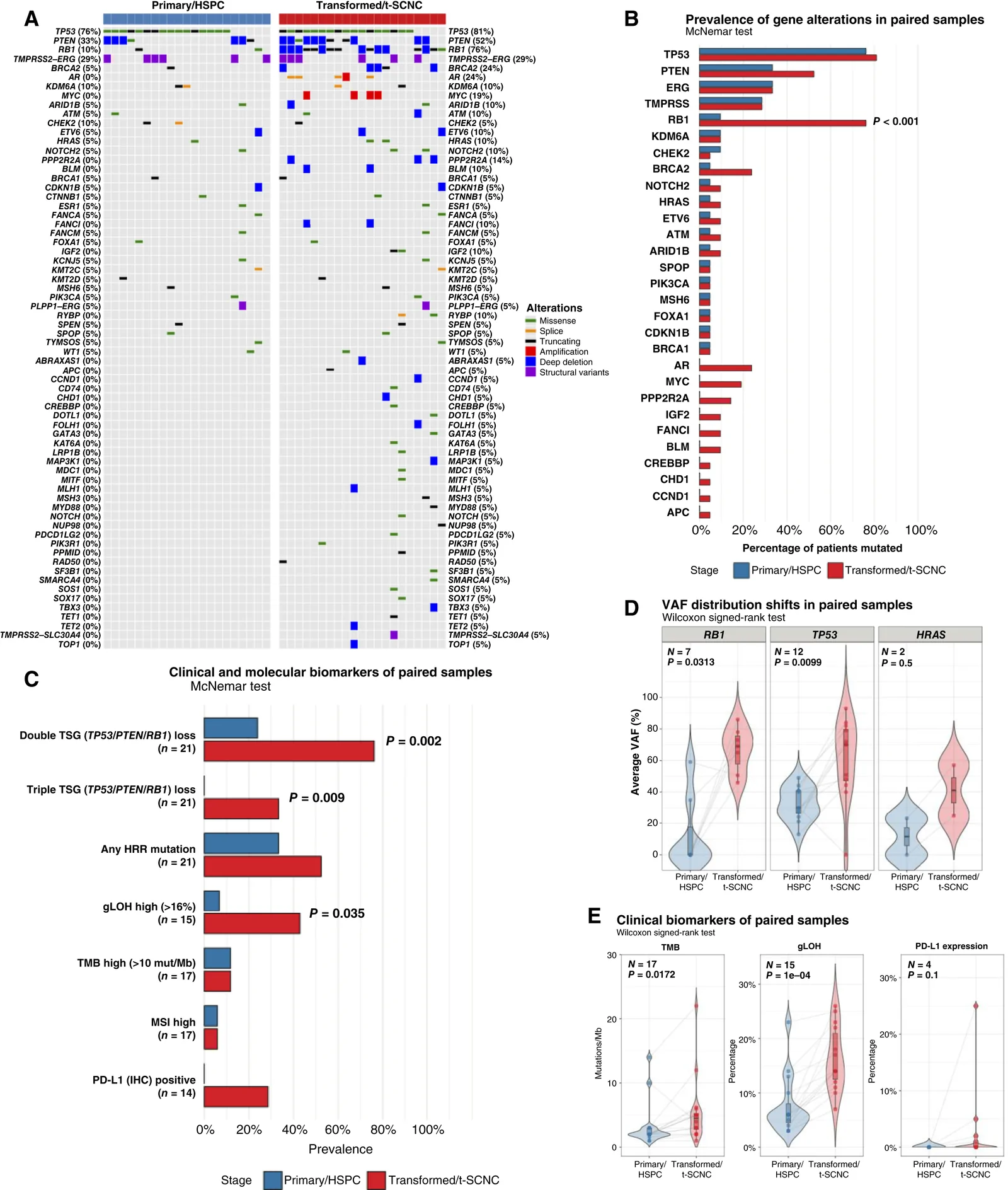
Genomic evolution in paired baseline and treatment-emergent tumor specimens. Genomic alterations were evaluated in 21 patients with paired baseline adenocarcinoma and treatment-emergent tumor specimens. **A,** OncoPrint depicting genomic alterations in paired baseline and treatment-emergent specimens. **B,** Prevalence of individual gene alterations in baseline and treatment-emergent specimens. **C,** Prevalence of aggregate molecular features, including double and triple TSG loss, HRR alterations, gLOH, TMB, microsatellite instability (MSI), and PD-L1 expression. McNemar test was used for paired comparisons of categorical features. **D,** VAFs of individual genomic alterations in paired baseline and treatment-emergent specimens. **E,** Paired comparison of quantitative molecular features, including TMB, gLOH, and PD-L1 expression. Wilcoxon signed-rank tests were used for paired continuous–variable comparisons.

To capture potential molecular patterns that may exist as part of the resistance pathway associated with the development of t-SCNC, we focused our analysis on the 21 patient-matched samples (Fig. 4A). We interrogated the prevalence of gene alterations, which were defined as genes harboring a missense mutation, splice variant, truncating mutation, amplification, deep deletion, or structural variant. Here, we found that the prevalence of *TP53* alterations was maintained between the pretreatment adenocarcinomas and the t-SCNC tumors (76%–81%) but observed more *PTEN* and *RB1* alterations in the t-SCNC tumors (33%–52%, 10%–76%, respectively). However, of these, only *RB1* alterations were significant (Fig. 4B), even when we examined the type of alteration event (Supplementary Fig. S3). Interestingly, although not significant, the 21 variant histology tumors also harbored increases in alterations in *AR* (0%–24%), *MYC* (0%–19%), and *BRCA2* (5%–24%). Here, we also examined aggregate changes. including double TSG loss, triple TSG loss, alterations in homologous recombination repair (HRR) genes, gLOH, TMB, microsatellite instability, and IHC of PD-L1 (Fig. 4C). Of these, double TSG loss, triple TSG loss, and gLOH-high were significantly increased in the t-SCNC tumors. Interestingly, among 14 evaluable paired samples, PD-L1 positivity increased in the t-SCNC specimens although this difference was not statistically significant. To examine potential changes in clonal representation and allele specificity, we evaluated VAF, which demonstrated that the VAFs of *RB1*, *TP53*, and *HRAS*, when observed, were all increased in the t-SCNC samples (Fig. 4D). This was also observed when we examined the measurements of TMB (mutations/Mb), gLOH (%), and PD-L1 expression (% positive; Fig. 4E). Altogether, in these paired samples, the findings support that loss of *RB1*, double or triple TSG loss, or gLOH was enriched in t-SCNC. This coincides with the pro-proliferative and unstable genomes associated with the aggressive tumor variants.

### Survival outcomes based on molecular features

**Figure 5.**
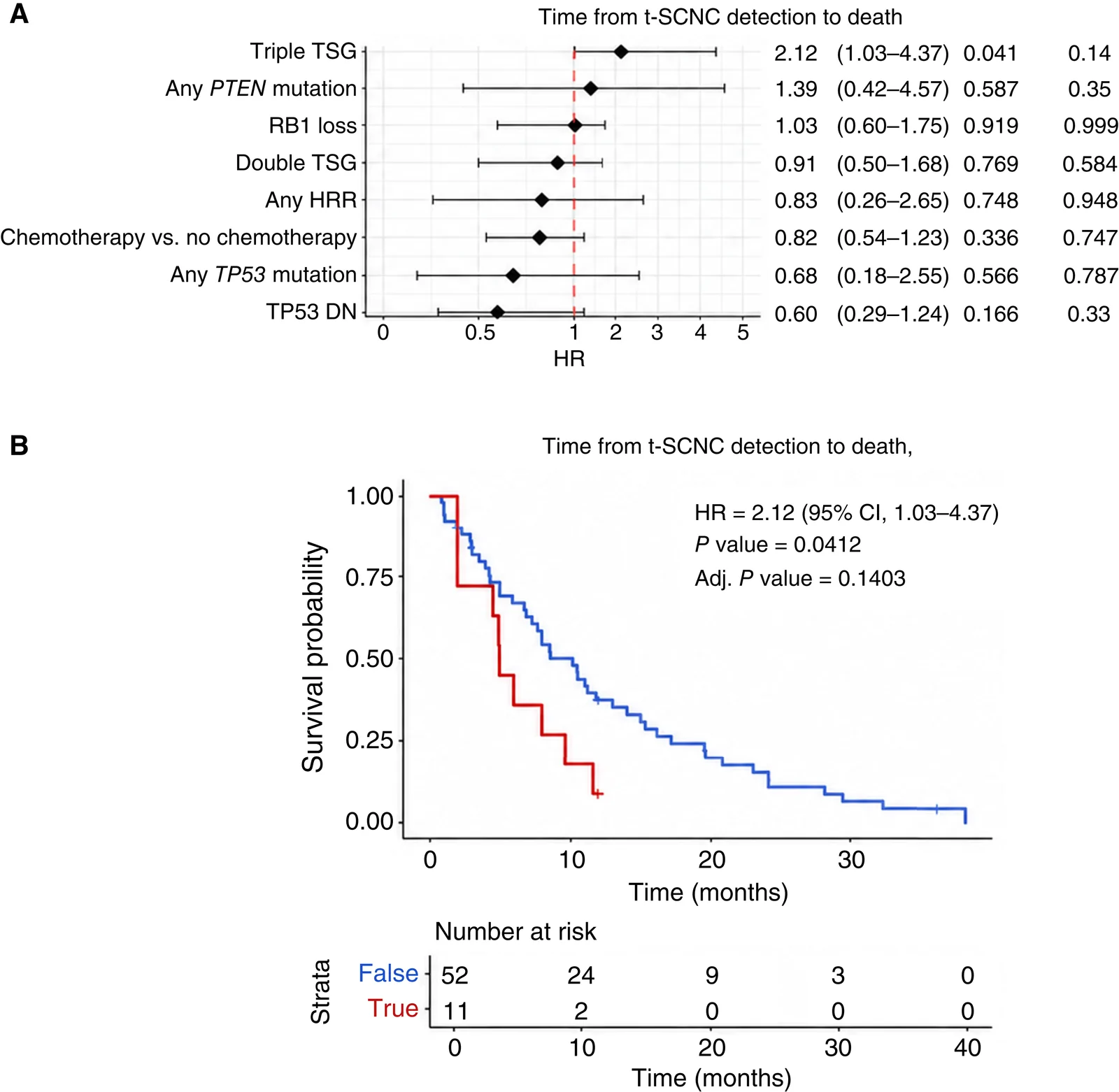
Clinical and molecular factors associated with OS following t-SCNC detection. **A,** Cox proportional hazards analysis evaluating clinical and molecular factors associated with OS following t-SCNC detection, including double and triple TSG loss, RB1 alterations, HRR alterations, TP53 alterations, DN TP53 alterations, and receipt of chemotherapy following t-SCNC detection. **B,** Kaplan–Meier estimates of OS following t-SCNC detection according to triple TSG loss (“true” delineating presence of triple TSG loss, “false” delineating absence of triple TSG loss).

To assess if certain genomic aberrations were associated with poor OS at t-SCNC detection, we evaluated whether the time to death was associated with the suite of molecular features (from t-SCNC specimens), including triple TSG loss, double TSG loss, *RB1* loss, HRR alterations, any *TP53* mutation, or DN *TP53* alterations. Given that a significant proportion of patients received first- or second-line chemotherapies after t-SCNC detection, we also evaluated the impact of this on survival as well (Fig. 5A). In univariate models, only triple TSG loss exhibited significantly worse outcomes (HR, 2.12; CI, 1.03–4.37; *P* = 0.041) after t-SCNC detection (Fig. 5B); this result did not remain significant in multivariable-adjusted models. There was a trend toward improved survival in patients who received cytotoxic chemotherapy after t-SCNC emergence (HR, 0.82; 95% CI, 0.54–1.23), but this did not meet significance.

## Discussion

The expanding armamentarium of systemic therapies in mHSPC—including earlier use of AR pathway inhibitors, chemotherapy, and other therapies such as radiopharmaceuticals—has altered the treatment landscape preceding the emergence of lineage-plastic disease. t-SCNC and other variant histologies are increasingly recognized as clinically important resistance states in advanced prostate cancer. Within this selected cohort, higher primary tumor grade was associated with a shorter interval from mHSPC diagnosis to t-SCNC detection in univariate analysis but not after multivariable adjustment, whereas prior chemotherapy and ADT plus ARPI exposure remained associated with shorter intervals to detection after adjustment. These findings should not be interpreted as evidence that these therapies increase the risk of transformation. Treatment exposure is influenced by disease aggressiveness, progression rate, treatment indication, and duration of disease, and patients with more aggressive clinical features may both receive more intensive therapy and undergo metastatic biopsy earlier. Advances in biopsy techniques have also made metastatic biopsies safer and more feasible. Consequently, confounding by indication and ascertainment bias may contribute to the observed associations. Our findings, therefore, characterize treatment histories and the relative timing of t-SCNC detection within this selected cohort rather than the causal effect of systemic therapy on transformation. Thus, there is a critical need to understand the potential predisposing biology of the parent tumor and to identify robust clinical and molecular predictors.

The number of t-SCNC cases identified in this study should not be interpreted as an estimate of its incidence or prevalence. In the prospective West Coast Prostate Cancer Dream Team study, Aggarwal and colleagues (2) enrolled consecutive patients with progressive mCRPC who underwent protocol-directed metastatic tumor biopsy, with t-SCNC identified in approximately 17% of patients. In contrast, our study retrospectively ascertained patients with pathologically confirmed treatment-emergent variant histology across participating institutions and did not include a denominator comprising all patients with mCRPC or all patients undergoing metastatic biopsy during the study period. Therefore, the number of cases identified across participating centers reflects retrospective ascertainment and clinical biopsy practices rather than the underlying prevalence of t-SCNC. Prostate cancers in which t-SCNC is subsequently detected may harbor distinct baseline molecular features that warrant further investigation. Our molecular studies identified a disproportional frequency of *TP53* alterations in the baseline samples, whereas *RB1* alterations were increased fivefold in the t-SCNC samples. With respect to *TP53* alterations, these rates were substantially higher compared with prior studies that selected for patients with mCRPC (25, 27). In the SU2C study of 444 mCRPCs, the rates of *TP53* alterations were at 40%, whereas this was 74% in the baseline samples of the cohort reported herein. The robust and consistent increase in VAFs of *TP53* alterations suggests an expansion of preexisting resistant subclones and/or progression to biallelic inactivation. Altogether, tumors that ultimately have t-SCNC detected may be distinguished less by broad genomic instability at presentation than by early enrichment for a *TP53*-centered tumor suppressor program. Logistically, it is a challenge to acquire genomically tested primary samples at diagnosis, which preceded treatment-emergent histology detection by a median of 33.9 months in this cohort. Therefore, expanding and validating these observations in prospective studies remains relevant. These findings raise the hypothesis that baseline *TP53* alterations may identify a population enriched for subsequent t-SCNC; however, this requires validation in prospective studies with an appropriately matched control population. Notably, no pathogenic CDK12 alterations were identified in our cohort. Although *CDK12* alterations have been associated with an aggressive clinical phenotype and adverse outcomes in prostate cancer (29), they seem to be relatively uncommon in neuroendocrine/small cell prostate cancer (30). Thus, their absence in our cohort may, in part, reflect sample size but is also consistent with the relative rarity of *CDK12* alterations in this disease phenotype.

Our findings are consistent with results observed from preclinical mouse models, in which genetic depletion of *Trp53* and *Rb1* were initiating events that were associated with the development of a small cell neuroendocrine phenotype (31). However, in clinical cases, our genomic data may support a stepwise model although it is possible that uncommon *RB1* loss clones are present at diagnosis but not detected by tumor genomic testing. Against a background of frequent baseline *TP53* alterations, we observed a fivefold increase in the detection of *RB1* disruptions in the t-SCNC specimens. In the paired patient samples, *RB1* alterations occurring in SCNC were associated with a significantly elevated VAF. This pattern is consistent with, but does not establish, later acquisition and/or clonal expansion during disease evolution. At the composite biomarker level, this culminated in significant increases in double *TP53*/*RB1* loss, triple *TP53*/*PTEN*/*RB1* loss, and gLOH-high status, consistent with a pattern of molecular evolution from adenocarcinoma to lineage-plastic disease. Interestingly, although AR activity may be lost, we did observe increases in AR alterations in the paired SCNC samples (0%–24%). This may not be entirely surprising, as prior studies have indicated that significant epigenetic reprogramming occurs in SCNC (2, 3, 16). Other studies have implicated lineage regulating transcription factors such as *SOX2*, *ASCL1*, *MYCN*, and *POU3F2* (18–21) and kinases such as *AURKA* and *RET* (22, 23) in driving or maintaining a small cell neuroendocrine lineage. Unfortunately, our genomic assessments are unable to evaluate the relative importance of these factors within disease progression, and this may require orthogonal assays that measure RNA or protein in these very specimens. However, spatial tumor heterogeneity represents an important consideration in the interpretation of these findings. Multiregion sequencing of prostate cancer has demonstrated that reliance on a single tissue region can incompletely represent dominant genomic alterations because of spatial heterogeneity and variable tumor content (32). Similarly, extensive rapid autopsy sampling of metastatic prostate cancer has demonstrated substantial intermetastatic phenotypic heterogeneity, including the coexistence of distinct AR/neuroendocrine-defined states within individual patients (33).

An important consideration is that treatment-emergent NEPC and other prostate cancers with lineage plasticity encompass a spectrum of histologic and phenotypic states rather than a single uniform morphologic entity. Our retrospective secondary pathology review identified tumors ranging from classical small cell carcinoma to other high-grade neuroendocrine carcinomas without definitive classical small cell morphology, adenocarcinomas with neuroendocrine features or a neuroendocrine component, amphicrine tumors, and other variant morphologies. These categories may represent overlapping but biologically distinct states of lineage plasticity, and their boundaries and clinical significance remain incompletely defined. The present study was not designed to determine whether these histopathologic subgroups differ in molecular evolution, treatment response, or prognosis. Prospective studies incorporating centralized pathology review and integrated morphologic, immunophenotypic, and molecular characterization will be required to refine this taxonomy.

No validated clinical risk stratification score is currently available in routine practice to predict the likelihood of t-SCNC in prostate cancer. Although several studies have developed gene expression signatures and transcriptional classifiers that can retrospectively identify or predict the emergence of SCNCs, these tools remain investigational and are not yet incorporated into clinical guidelines or standard risk models (3, 16). The National Comprehensive Cancer Network and the American Urological Association guidelines do not endorse any specific risk score for this purpose. Clinical features previously associated with lineage plasticity and t-SCNC include high initial tumor grade (grade group 5/Gleason score 9–10) and prior exposure to potent ARPIs (3, 18, 19). Consistent with these prior observations, ARPI exposure was associated with a shorter interval from mHSPC diagnosis to t-SCNC detection within our selected cohort; however, the present study cannot determine whether ARPI exposure independently increases the risk of transformation.

There are no established guideline-endorsed screening strategies for the early detection of t-SCNC or other variant histologies in prostate cancer; however, expert recommendations have been published in the literature (10). In routine practice, biopsy is generally pursued when the clinical phenotype becomes discordant with typical adenocarcinoma behavior, such as rapid progression, visceral disease, lytic lesions, or progression out of proportion to PSA increase (34). Our results reinforce this challenge: Despite substantial disease burden at the time of treatment-emergent histology detection, the median PSA remained relatively low, and only 33.9% of patients with evaluable PSA trajectory demonstrated a >25% increase above PSA nadir. Conversely, approximately two thirds had stable or declining PSA at the time of variant histology detection. This discordance between PSA kinetics and disease evolution is consistent with reduced dependence on AR signaling in treatment-emergent neuroendocrine disease and highlights the limitations of PSA alone as a marker of disease progression in this setting. Imaging modalities, including conventional CT and bone scans, are not specific for the emergence of small cell histology. Neuroendocrine histology can be associated with a reduction in prostate-specific membrane antigen levels and an augmented expression of somatostatin receptors (35, 36). The systematic review by Dondi and colleagues (37) surveyed studies evaluating multiple nuclear imaging modalities, indicating that PET/CT with fluorodeoxyglucose or somatostatin analogs may prove most helpful in this setting. However, these implementations may require substantial development in a minor population of patients with prostate cancer.

Optimal first-line treatment for t-SCNC is generally accepted to be platinum-based chemotherapy with either cisplatin or carboplatin plus etoposide (12), as well as the utilization of immunotherapy. This recommendation is extrapolated from the management of small cell lung cancer due to the lack of large prospective trials in prostate cancer. For patients with deleterious alterations in two or more TSGs (such as *TP53* and *RB1*), ASCO suggests considering carboplatin plus cabazitaxel although this is supported by lower-quality evidence (12). The survival analyses were limited by sample size; aside from triple TSG loss in univariate analysis, no evaluated molecular feature was significantly associated with OS following t-SCNC detection.

Although our findings support further investigation of molecular features that may identify adenocarcinomas enriched for subsequent t-SCNC, such as combined *TP53* and *RB1* inactivation or accumulating tumor-suppressor gene alterations, there are currently no interventions that effectively suppress lineage plasticity or prevent evolution to t-SCNC. This represents an important unmet clinical need. Circulating assays, including cell-free DNA methylation and circulating tumor cell–based platforms, are increasingly able to detect treatment-emergent neuroendocrine/small cell features noninvasively; however, these approaches have not yet been prospectively validated as treatment-switching tools that improve clinical outcomes. After progression on platinum-based chemotherapy, enrollment in clinical trials is recommended, as there is no established second-line standard of care. Other options, such as lurbinectedin, topotecan, or immunotherapy, may be considered on a case-by-case basis, but evidence is limited. Based on the phase Ib/II KEYNOTE-365 study (NCT02861573), the addition of pembrolizumab to chemotherapy with carboplatin and etoposide may be associated with improved efficacy compared with chemotherapy alone. The median radiographic progression-free survival was 5.1 months (95% CI, 3.9–8.1) with pembrolizumab plus chemotherapy compared with 4 months (95% CI, 2–4.3) with chemotherapy alone (38). The phase I study of the bispecific T-cell engager tarlatamab in patients with aggressive variant prostate cancer, including t-SCNC, demonstrated manageable safety with encouraging antitumor activity in DLL3-expressing cases (15); however, further investigation is ongoing. More recently, the C3NIRA (14) and CHAMP (39) trials have also reported encouraging activity with immunotherapy-based combination strategies in aggressive variant prostate cancer and NEPC although further randomized studies are needed.

### Study limitations

These findings should be interpreted in the context of several cohort study limitations. This retrospective cohort was assembled from tertiary academic centers over a period extending several years, during which the mHSPC and mCRPC treatment paradigms evolved substantially. These factors may introduce potential referral and selection biases that should be considered. Consistent with this extended treatment era, only 25% of patients received upfront ADT plus ARPI doublet therapy, and 4.3% received upfront triplet therapy, limiting the generalizability of treatment-exposure patterns to patients treated under contemporary mHSPC standards. The absence of centralized pathology review should also be considered a limitation, as primary histologic classifications were based on diagnoses rendered at the participating institutions. Furthermore, IHC markers, AR/PSA expression, Ki-67, and mitotic rate data were not uniformly available across all specimens, limiting the retrospective application of a single standardized histopathologic definition. In addition, NGS assays differed across institutions, and genomic alterations were derived from clinical reports rather than uniformly reprocessed sequencing data. Although all genes and alteration classes included in Figs. 2–4 were evaluable by the assays contributing to those analyses, differences in analytic sensitivity between platforms, particularly for copy-number alterations and structural variants/fusions, remain a limitation. Comparisons with external cohorts, including TCGA and Mateo and colleagues, should also be interpreted cautiously because of differences in disease stage, cohort selection, specimen timing, and sequencing platforms; in the absence of a matched control population, the observed baseline *TP53* enrichment cannot establish *TP53* alterations as a predictor of subsequent t-SCNC. Not all patients included had available NGS data at baseline or at the time of t-SCNC, and only 21 cases with paired biopsies were available for the assessment of within-patient genomic evolution. Importantly, this study lacked a comparator group of patients receiving similar systemic therapies who did not develop t-SCNC. Treatment exposures were also time-dependent and influenced by disease aggressiveness, progression rate, treatment indication, duration of disease, and evolving standards of care. Patients with more aggressive disease may have received more intensive systemic therapy and may also have undergone metastatic biopsy earlier, introducing confounding by indication and ascertainment bias. Accordingly, the observed associations between prior treatment exposure and time to t-SCNC detection should be interpreted as within-cohort associations with the timing of detection and do not establish that treatment intensification increases the incidence or risk of transformation to t-SCNC. Finally, identification of treatment-emergent variant histology was based on single-site tissue biopsies, which may not fully capture intrapatient spatial heterogeneity. Multiregion sampling studies have demonstrated that the analysis of a single tumor region can incompletely represent the dominant prostate cancer genotype, raising the possibility that coexisting histologic or molecular subclones may not have been detected in the sampled lesion.

### Summary

t-SCNC remains an aggressive disease entity with poor outcomes. Our large multi-institutional cohort, with paired molecular specimens available for a subset of 21 patients, may support the concept of transdifferentiation from prostate adenocarcinoma to a variant histology under selective treatment pressure, marked by convergent inactivation of *TP53*, *RB1*, and *PTEN*, with divergence in the timing and completeness of *RB1* loss between histologies. Our aggregate findings reinforce the urgent need for prospective biomarkers of t-SCNC evolution, refined detection strategies, and tailored therapeutic approaches, including platinum-based chemotherapy, immunotherapy, and clinical trial enrollment for patients with treatment-emergent small cell or other aggressive variant prostate cancers.

## Supplementary Material

Supplementary Table S1 Table S1. Biopsy sites, histopathologic classification, PSA trajectory, and genomic testing platforms.

Supplementary Table S2 Table S2. Time to treatment-emergent histology and detailed treatment history.

Supplementary Figure S1 Figure S1. PSA dynamics and time to treatment-emergent variant histology.

Supplementary Figure S2 Figure S2. Genomic alteration types in baseline and treatment-emergent tumor specimens.

Supplementary Figure S3 Figure S3. Genomic alteration types in paired baseline and treatment-emergent tumor specimens.

Supplementary Figure S4 Figure S4. Dominant-negative TP53 alterations across baseline and treatment-emergent tumor cohorts.

## Data Availability

In this study, we analyzed deidentified clinical data collected retrospectively across participating tertiary academic centers, which is subject to controlled access due to institutional policies that may differ across the institutions that have participated in this study. Deidentified molecular testing data underlying the reported analyses may be made available upon reasonable request to the corresponding authors, subject to applicable institutional data use agreements.
